# EHMT2/G9a as an Epigenetic Target in Pediatric and Adult Brain Tumors

**DOI:** 10.3390/ijms222011292

**Published:** 2021-10-19

**Authors:** Barbara Kunzler Souza, Natalia Hogetop Freire, Mariane Jaeger, Caroline Brunetto de Farias, Algemir L. Brunetto, André T. Brunetto, Rafael Roesler

**Affiliations:** 1 Cancer and Neurobiology Laboratory, Experimental Research Center, Clinical Hospital (CPE-HCPA), Federal University of Rio Grande do Sul, Porto Alegre 90035-003, Brazil; nfreire@hcpa.edu.br (N.H.F.); labpesquisa1@ici.ong (M.J.); labpesquisa@ici.ong (C.B.d.F.); brunettoa@hotmail.com (A.L.B.); andrebrunetto@ici.ong (A.T.B.); 2 Epigenica Biosciences, Canoas 92035-000, Brazil; 3 Children’s Cancer Institute, Porto Alegre 90620-110, Brazil; 4 Department of Pharmacology, Institute for Basic Health Sciences, Federal University of Rio Grande do Sul, Porto Alegre 90050-170, Brazil

**Keywords:** G9a, EHMT2, glioblastoma, medulloblastoma, epigenetics, brain tumor

## Abstract

Epigenetic mechanisms, including post-translational modifications of DNA and histones that influence chromatin structure, regulate gene expression during normal development and are also involved in carcinogenesis and cancer progression. The histone methyltransferase G9a (euchromatic histone lysine methyltransferase 2, EHMT2), which mostly mediates mono- and dimethylation by histone H3 lysine 9 (H3K9), influences gene expression involved in embryonic development and tissue differentiation. Overexpression of G9a has been observed in several cancer types, and different classes of G9a inhibitors have been developed as potential anticancer agents. Here, we review the emerging evidence suggesting the involvement of changes in G9a activity in brain tumors, namely glioblastoma (GBM), the main type of primary malignant brain cancer in adults, and medulloblastoma (MB), the most common type of malignant brain cancer in children. We also discuss the role of G9a in neuroblastoma (NB) and the drug development of G9a inhibitors.

## 1. Introduction

The concept of epigenetic regulation comprises heritable and non-heritable long-term changes in gene expression that are not dependent on mutations in DNA sequences. Epigenetic mechanisms that regulate gene expression, such as post-translational modifications of DNA and histones influencing chromatin structure, are now established players in cancer biology. Chromatin is organized in units called nucleosomes, each of which consist of a 146 bp segment of DNA wrapped around an octamer of histone proteins. Histone acetylation and deacetylation, and histone and DNA methylation act in concert to regulate the relaxation status of chromatin and transcriptional activity [1]. In cancer, chromatin condensation and DNA methylation can favor the repression of genes that promote differentiation and cell death, while promoting expression of genes related to cell survival, proliferation, and stemness. This reflects the view of cancer as a developmental disease, where carcinogenesis hijacks biological processes involved in normal embryonic development. Epigenetic programming orchestrates the different phases of tissue development, mediating the transition—together with growth factors and other biochemical signals—from a highly proliferative and non-differentiated state to later stages where differentiation and cell selection become key for establishing specialized cells and tissues from an original pluripotent cell. In cancer, the epigenetic machinery can be maintained in a state that keeps a less differentiated, stem cell-like, and highly proliferative phenotype. Thus, experimental epigenetic therapies are currently investigated with the aim of reverting the oncogenic epigenetic patterns of histone modifications and DNA methylation that maintain tumor malignancy [2,3,4]. In brain tumors and other tumor types possibly originating from neuroectodermal or neural stem cells, abnormal epigenetic programming may stimulate cell survival and stemness, and epigenetic compounds can increase cell death and promote neural differentiation [5,6,7,8,9,10]. 

The best-studied epigenetic modification in cancer is DNA methylation. It results in condensed chromatin structure and transcriptional inactivation, thus repressing or silencing genes. DNA methyltransferases (DNMTs) mediate DNA methylation that occurs mostly in DNA regions called “CpG islands”, which concentrate CpG dinucleotides at gene promoters, regulatory regions, and gene bodies [11]. However, increasing attention has also been paid to histone methylation, which is mainly regulated by histone methyltransferases (HMTs). Methylation, as well as acetylation, phosphorylation, ubiquitination, and ADP-ribosylation, modify the N-terminal tails of core histones, resulting in relaxation or condensation of chromatin due to changes in the affinity between histones and DNA, or regulating gene expression by interfering with the binding of transcription factors to DNA sequences [12,13]. Histone methylation is a reversible post-translational modification where up to three methyl groups can be added to a histone lysine residue and up to two groups to a histone arginine residue, resulting in an epigenetic mark that can either activate or repress overall levels of gene expression [14]. 

**Figure 1:**
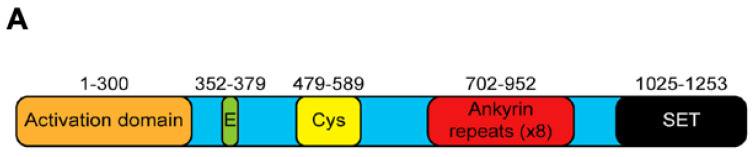
Structure of G9a. The protein contains 1253 amino acids and distinct domains including an *N*-terminal activation domain, glutamate-rich (23 consecutive Glu residues), and cysteine-rich regions, eight ankyrin repeat units (binding of dimethylated lysine residues), and a C-terminal enzymatic SET domain [23].

Methylation of H3 lysine 9 (H3K9) is a hallmark of heterochromatin, the condensed, transcriptionally inactive state of chromatin. Mono- and dimethylation of H3K9 are mediated by the HMT G9a (euchromatic histone lysine methyltransferase 2, EHMT2) [15]. G9a is encoded by the *Ehmt2* gene, located in the major histocompatibility complex (MHC) locus in mice and human leukocyte antigen (HLA) locus in humans, and it contains 28 exons that code for a 1263 amino acid nuclear protein belonging to the Su(var)3-9 family [16,17]. The different domains comprising G9a are a catalytic SET domain, a domain containing ankyrin repeats involved in protein–protein interactions, and nuclear localization signals on the *N*-terminal region (Figure 1). However, G9a does not contain a DNA-binding domain, requiring cofactors for its localization to specific genes [18,19,20,21,22,23,24]. Through its methyltransferase activity, as well as methyltransferase-independent actions mediated by its *N*-terminal domain [23,25,26], G9a regulates changes in gene expression involved in embryonic development and differentiation of normal tissues [19,20,21,24].

Overexpression of G9a is found across different solid tumor types, including lung, ovarian, esophageal, hepatocellular, and brain cancers, as well as in multiple myeloma, and it has been associated with poor prognosis in several cancer types [27,28,29]. Higher G9a levels are associated with increased methylation that inhibits the expression of tumor suppressor genes, likely resulting in more aggressive phenotypes, with increased invasiveness and metastasis [30,31,32]. For example, small interfering RNA (siRNA)-mediated G9a knockdown rescues the expression of the tumor-suppressor gene MASPIN in MDA-MB-231 breast cancer cells [31]. Expression of G9a is higher in metastatic lesions compared to their corresponding primary tumors in ovarian cancer, and knockdown of G9a inhibits prometastatic cellular activities while G9a over-expression promotes these cellular properties [30]. In this review, we will focus on the role of G9a as an epigenetic regulator and pro-tumoral controller in brain tumors.

## 2. G9a and Glioma

Glioblastoma (GBM) is the most aggressive type of primary malignant brain tumor in adults, and also presents pediatric types, such as diffuse intrinsic pontine glioma and pediatric non-brainstem high-grade glioma. GBM can be a primary tumor or develop as secondary GBM from lower-grade tumors harboring a mutation in isocitrate dehydrogenase (IDH). Current treatment based on combining surgical resection followed by radiotherapy and chemotherapy results in a median overall survival of less than 2 years, and the development of novel, molecularly targeted therapies is urgently needed [33,34,35,36]. It has been less than 10 years since the first evidence of a role for G9a in GBM and gliomas of lower grades started to emerge. H3K9me3, a marker of G9a-mediated repression of transcription, is found across different types of astrocytic tumors (48% of pilocytic astrocytomas, 78% of diffuse astrocytomas, 67% of anaplastic astrocytomas, and 79% of GBMs) and is significantly associated with IDH mutations in oligodendrogliomas. Combined H3K9me3 positivity and 1p19q codeletion is found in most World Health Organization (WHO) grade II and grade III oligodendrogliomas. H3K9me3 is a prognostic marker of improved overall survival in grade II oligodendrogliomas compared with H3K9me3-negative cases. These findings suggest that H3K9me3 may define a subset of tumors with better overall survival in grade II oligodendrogliomas, but not in higher grade gliomas [37]. Thus, G9a may have a complex role where, at least in some selected cancer types, its expression is related to a better rather than worst prognosis.

Changes in expression of genes coding for lysine and arginine methyltransferases including G9a play a role in GBM pathogenesis [38]. An immunohistochemical study aimed at investigating G9a, H3K9me2 and histone H3K9me1 in human glioma and adjacent non-neoplastic tissue samples found that 86% (43 of 50) are positive for G9a expression, compared to 42% (21 of 50 samples) in non-neoplastic tissues. Expressions of G9a and H3K9me2 are positively associated with a higher WHO glioma grade and the G9a inhibitor BIX-01294 inhibits proliferation, induces apoptosis, and reduces mono- and dimethylation of H3K9 in U251 human GBM cells [39]. 

A network of epigenetic components including G9a regulates gene expression in GBM cells. G9a can form a protein complex with a DNMT to epigenetically regulate the *NY-ESO1* gene, which is a cancer/testis antigen proposed as a suitable target for cancer immunotherapy [40]. G9a is highly expressed in GBM cells, and pharmacological inhibition or genetic knockdown of G9a reduces cell proliferation and tumorigenesis in experimental GBM in vitro and in vivo. Knockdown of G9a also results in cell cycle arrest in the G2 phase accompanied by reduced expression of CDK1, CDK2, Cyclin A2, and Cyclin B1 and activation of autophagy [41]. The G9a inhibitor BIX01294 triggers apoptosis and autophagy, increases apoptosis markers cleaved caspase 3, caspase 7, and poly (ADP-ribose) polymerase (PARP), and reduces H3K9 dimethylation (H3K9me2) in cultured GBM cells, in addition to promoting differentiation of GBM stem cells [42]. The same inhibitor also sensitizes GBM cells to the effects of the cytotoxic chemotherapeutic temozolomide, in terms of reduced cell viability, apoptotic-like alterations in morphology, and increase in apoptosis markers [43,44], in addition to radiosensitizing GBM cells and inhibiting DNA double-strand breaks through both the homologous recombination and nonhomologous end-joining pathways [45].

Both pharmacological inhibition and siRNA-mediated knockdown of G9a increases the content of the autophagy marker LC3B in GBM cells [46]. G9a and the related methyltransferase protein, GLP, directly bind to the α subunit of HIF-1 (HIF-1α) and catalyze mono- and dimethylation of HIF-1α at lysine (K) 674 in vitro and in vivo [47]. The effects of G9a are at least partially mediated by the long non-coding RNA HOTAIRM1, which transcribes from the antisense strand of HOXA gene cluster which locus in chromosome 7p15.2. HOTAIRM1 functions as an oncogenic factor regulating HOXA1 gene expression and sequestering G9a and DNMTs away from the HOXA1 gene promoter [48]. 

In tumors, epigenetic programming is crucial for maintenance of a stem-cell phenotype, with high proliferation but inhibited differentiation. Interferon γ (IFNγ) increases H3K9me2 and reduces G9a levels, whereas G9a inhibition increases IFNγ while reducing retinoic acid inducible gene (RIG-I) in GBM cells. These findings indicate a concerted interplay between G9a and the PPAR gamma coactivator-1 alpha (PGC-1α) to promote RIG-I-induced maintenance of a stem cell-like state in GBM cells [49]. One study [50] found that most GBM cells positive for expression of the stem cell marker CD133 are negative for G9a-dependent H3K9me2. Pharmacological inhibition of G9a stimulates sphere formation (an index of cancer stem cell expansion in vitro) and increases expression of stem cell markers CD133 and Sox2 in GBM cell cultures. Conversely, overexpression of G9a increases H3K9me2 and reduces sphere formation along with CD133 and Sox2 content. Moreover, the authors confirmed that H3K9me2 acts on CD133 and Sox2 promoter regions to repress their expression. Together, these findings strongly indicate that, although G9a may function as a promoter of brain tumor progression through maintenance of a stem cell-like phenotype, at least under some conditions it can also inhibit brain tumor stem cell formation. Expression of genes related to autophagy and differentiation is upregulated by G9a inhibition via BIX01294 in putative glioma stem cells from spheres [42]. BIX01294 enhances the cytotoxic effects of temozolomide in GBM stem-like cells, even in the absence of changes in pluripotency markers NANOG, SOX2, and CD133, or methylation of NANOG and SOX2 gene promoters. These results suggest a potential role for G9a inhibition as a component of a combination therapy strategy to increase the efficacy and reduce resistance to cytotoxic chemotherapeutics [43].

## 3. G9a and Medulloblastoma

In children, brain tumors are the most common solid tumors and the leading cause of cancer-related death. Medulloblastoma (MB) is the most common type of malignant brain cancer in childhood and can rarely occur in adults as well. Remarkable advancements in our understanding of MB biology have been made during the past decade. The current molecular classification divides MB tumors into four consensus subgroups with distinct genomic, epigenetic, and clinical features: WNT, SHH, Group 3, and Group 4 [51,52]. This molecular classification has rapidly made its translation to the clinical setting, becoming important for guiding patient risk stratification, treatment, and selection in clinical trials [53,54]. Two subgroups are defined by mutations leading to aberrant activation of the Wingless (WNT) and Sonic hedgehog (SHH) pathways, whereas Group 3 MB display amplification of genes involved in the Notch and transforming growth factor-β (TGFβ) pathways, and Group 4 show a prominent representation of genes involved in regulating chromatin state [51,55]. Group 3 and Group 4 MBs are more aggressive and associated with a poor prognosis, with patients with Group 3 MB often showing metastasis at diagnosis and having a 5-year survival of around 50% [53,55,56]. Recent descriptions of intra- and intertumoral cellular and molecular heterogeneity within subgroups have led to further classifying MB into 12 subtypes. Although multimodal treatments with surgery radiotherapy and chemotherapy has improved cure rates, about one-third of patients relapse, and survivors experience long-lasting neurological, cognitive, and endocrinological sequelae. Thus, the development of molecularly targeted therapies is urgently needed [55,56,57,58]. 

The deubiquitylase USP37 regulates cell proliferation by acting on the stability of the cyclin-dependent kinase inhibitor 1B (CDKN1B/p27Kip1) and may act as a tumor suppressor in MB. Expression of USP37 is downregulated in human MB, and USP37 reduces the growth of experimental MB. G9a reduces USP37 expression by promoting H3K9 mono-, di-, and trimethylation at its promoter. The USP37 promoter shows a considerable level of H3K9 trimethylation, which is reduced after inhibition of G9a activity. These findings reveal a role for G9a as an epigenetic regulator of G9a MB. In addition, the G9a inhibitor UNC0638 impairs proliferation of human DAOY MB cells and MB growth in vivo through mechanisms that include downregulating USP37 [59,60].

G9a shows interplays with another histone methyltransferase, EZH2, often constituting an “axis” of functionally related epigenetic modifiers influencing cancer cells. EZH2 has been shown to play a role in GBM and MB, and EZH2 inhibitors have been evaluated in preclinical models of brain cancers. For example, the pyrazole compound MC3629, developed as an analog of two different SAM-competitive EZH2 inhibitors, EPZ005687 and GSK2816126, has been shown as an inhibitor of G91/EZH2 signaling in MB capable of impairing cell proliferation, stemness, and H3K27me3, and inducing apoptosis in human MB cell models representing the SHH group, as well as in a mouse model of SHH MB xenografts. Moreover, MC3629 showed good blood–brain barrier penetration and was able to reduce H3K27me3 levels in the brain and cerebellum in mice [61].

## 4. G9a and Neuroblastoma

Neuroblastoma (NB) arises commonly in the peripheral nervous system, from embryonal neural crest cells that later give rise to the sympathetic nervous system and is thus not commonly classified as a brain tumor [62,63,64]. However, it can also rarely occur intracranially [65,66,67] and will thus be included in the present review. NB accounts for around 15% of cancer-related deaths in children and adolescents. High-risk NB, usually defined by amplification of the MYCN oncogene, confers a poor prognosis with short overall survival [64]. NB shows an abnormal epigenetic reprogramming, with DNA methylation-induced repression of tumor suppressing genes [68,69].

A microarray-based search of tumors from 88 patients showed that low G9a expression is correlated with better overall survival, whereas, in contrast, high G9a expression is associated with a poor outcome. Expression of G9a varies with tumor stage, where expression is higher in stage 4 tumors compared to stages 3 and 4S. Furthermore, G9a expression is significantly higher in patients having the NB tumor as cause of death compared to the non-death group. Treatment of NB cell lines were treated with the G9a inhibitor BIX01294 resulted in a reduction in both cell proliferation in vitro and tumorigenicity in NOD/SCID mice, in addition to the appearance of markers of autophagy. Finally, similar findings are observed in cells submitted to G9a knockdown with lentiviral human G9a shRNA [70]. Treating human SH-SY5Y NB cells with the estrogenic endocrine disruptor bisphenol A led to a reduction in H3K9me3 and changes in expression of G9a among other chromatin modifying genes [71].

Immunological analysis showed G9a protein to be more highly expressed in NB cell lines harboring MYCN amplification, a crucial marker of poor outcome in NB patients. In primary NB tumors, the content of G9a protein is higher in poorly differentiated or undifferentiated tumors and correlates with expression of the related oncoprotein EZH2. G9a depletion via use of siRNA, or treatment with G9a inhibitors, UNC0638 and UNC0642, reactivates tumor suppressor genes, reduces NB cell proliferation and induces apoptosis, but these effects are observed selectively in cells with MYCN amplification. The need for MYCN for G9a inhibition to be effective is also demonstrated in the SHEP-21N isogenic model with tet-regulatable MYCN [72]. G9a and GLP are repressors of transcriptional responses to interferon-γ (IFN-γ) in NB cells. Inhibition of G9a and GLP enhances IFN-γ-induced expression of the Th1-type chemokines CXCL9 and CXCL10, which crucially mediate recruitment of T-cells to the tumor microenvironment. G9a inhibition is required for transcriptional responses to IFN-γ and histone mark changes at CXCL9 and CXCL10 gene loci in MYCN-positive NB cells. These findings highlight the important role of G9a in high-risk, MYCN-expressing, NB [73].

## 5. Drug Development of G9a Inhibitors for Cancer Therapy

The small molecule compound BIX01294, which displays inhibitory activity on both G9a and GLP, is an early inhibitor developed about a decade ago that represents a 2,4-diamino-6,7-dimethoxyquinazoline template. Building upon the same template, compound 10 (UNC0224) was developed as another G9a inhibitor with excellent selectivity and potency. Structural insight from the X-ray crystal structure of the G9a-10 complex enabled optimization of the 7-dimethylaminopropoxy side chain of UNC0224 resulted in the discovery of compound 29 (UNC0321), the first G9a inhibitor with potency at picomolar concentrations [74]. Further developments by the same group, for the design new generations of analogues aimed at improving cell membrane permeability, led to the development of UNC0646 and UNC0631, which show excellent potency in cells as well as excellent relationship between potency and cytotoxicity [75]. More recently, the 2-alkyl-5-amino- and 2-aryl-5-amino-substituted 3 H-benzo[e] [1,4] diazepine scaffold was used to identify compound 12a (EML741), which displays high in vitro and cellular potency comparable to BIX01294 inhibitory activity against DNMT1 [76]. Replacing the hydrophobic segment of BIX-01294 and UNC0638 with a guanidine moiety (side-chain moiety of arginine) resulted in additional compounds. The guanidine moieties were positioned similar to the Arg8 of the substrate peptide in molecular docking, and reactivity of the guanidine-substituted inhibitors was observed in density functional theory studies. Molecular dynamics, molecular mechanics Poisson–Boltzmann surface area binding free energy, linear interaction energy, and potential mean force calculated from steered molecular dynamics simulations showed improved conformational stability and improved H-bond potential and binding affinity of the new compounds. In conclusion, the authors propose incorporating a guanidine group to mimic the substrate arginine side chain and improve the potency of G9a inhibitors [77].

An alkaloid derived from Chaetomium minutum, chaetocin, which is a structurally complex epidithiodiketopiperazine (ETP) shown to be a potent G9a inhibitor. Synthetic approaches aimed at discovering other G9a inhibitors based on structure–activity relationships of chaetocin derivatives resulted in the novel derivative PS-ETP-1 [78]. Another alkaloid, protoberberine alkaloid pseudodehydrocorydaline (CT13) was identified as a novel G9a inhibitor by structure-based virtual screening of an in-house library containing natural products. The activity of CT13 was determined by mass spectrometry and Western blot analysis, and the compound displays selective inhibition of G9a in human breast cancer cells. Molecular docking indicated that CT13 acts on the histone H3 binding site [79]. 

Six novel analogs were designed with 3D quantitative structure–activity relationship (3D-QSAR) analysis of a series of 2,4-diamino-7-aminoalkoxyquinazoline G9a inhibitors. Structural requirements for substituted 2,4-diamino-7-aminoalkoxyquinazoline for G9a inhibitory activity could be obtained from comparative molecular similarity indices analysis (COMSIA) plots [80]. Through structure-based virtual screening, DCG066 was developed as a new G9a inhibitor with a molecular scaffold distinct from previous inhibitors. This compound binds G9a directly and inhibits its activity in vitro in addition to reducing H3 methylation [81]. CPUY074020, based on the structure of UNC0638 and containing a 6H-anthra[1,9-cd]isoxazol-6-one scaffold, was developed as a leading compound displaying potent dual G9a inhibitory and anti-proliferative activities, and it also promoted cell apoptosis and reduced dimethylation of H3K9. Moreover, this compound shows good pharmacokinetic properties in vivo [82]. Structure-based design, synthesis, and screening of small molecules for dual inhibitory activity of G9a and histone deacetylases (HDACs) allowed for the discovery of compound 14, which inhibits both enzymes in the low micro-molar range in cell-based platforms [83]. Structure-based approaches also led to the design and synthesis of reversible chemical probes that inhibit both G9a and DNMTs at nanomolar ranges, resulting in compounds with in vitro antiproliferative activities in the nanomolar range and tumor growth inhibition in a human acute myeloid leukemia mouse model [84].

Molecular dynamics simulation and free energy calculations of five different modified/mutated G9a substrate peptides, in addition to evaluation of the binding energy contribution-based architecture of the active site of G9a, was used to detail the molecular basis of G9a binding to inhibitors. These experiments revealed, for example, that Arg8 of the substrate peptide is crucial for determining binding to G9a. The G9a active site is segregated into energy-rich and low regions, and the energy-rich region is used for binding inhibitors. The findings suggested that potential G9a inhibitors would preferentially interact with residues Asp1074, Asp1083, Leu1086, Asp1088, Tyr1154, and Phe1158 og G9a [85]. Further structure-based analysis of activity cliffs, scaffold hops, and G9a inhibitors with docking followed by molecular dynamics simulations confirmed the identification of these key residues [86].

Recent advances include the discovery, through a virtual screening strategy, of CSV0C018875, a novel quinoline-based G9a inhibitor. Additionally, sub-structure querying based on previous G9a inhibitors together with docking-based virtual screening, led to the identification of CSV0C018875, which, according to molecular dynamics simulations, binds deeper inside the active site of G9a, likely enabling tighter binding and a longer time of action [87].

## 6. Conclusions and Future Directions

The treatment of brain tumors remains one of the most challenging unmet medical needs of our time. In the case of GBM, for example, the virtual absence of pharmacological options and the lack of curative treatment protocols, combined with the very poor prognosis with a short life expectancy after diagnosis, make it absolutely urgent to search for novel therapeutics. With MB, more effective and less toxic treatments are needed to improve the outcome of pediatric and adult patients with this tumor that represents one of the most important causes of disease-related death in children.

Given the extensive epigenetic reprogramming present in brain tumors, which redefines gene expression in an aberrant way to enhance cell proliferation, survival, and stemness while inhibiting apoptosis and differentiation, molecular components of epigenetic regulation have increasingly been highlighted as targets to help establish the malignant phenotype seen in these cancers. In comparison to genetic mutations and other changes in DNA sequence, the epigenome provides more druggable targets, given the difficulty of directly manipulating DNA with pharmacological agents. Furthermore, compared to more specific molecular targets related to cell signaling, such as growth factor receptors and protein kinases, manipulating epigenetic processes may result in broader changes in gene expression, altering transcription of several sets of genes, possibly leading to a larger modification of the cellular phenotype. Moreover, given the increasingly recognized intertumoral and intratumoral complexity in terms of genetic and biochemical heterogeneity [88], epigenetic drugs could hypothetically be able to produce effects in cancer cells and tumors that show differences in genetic mutations or activation of signaling pathways. Based on the previous experience with epigenetic modulators in experimental cancer, it is possible that, in various circumstances, combination therapies using one G9a inhibitor plus cytotoxic chemotherapy, other epigenetic agents, or other classes of targeted agents will be revealed as more effective than single-agent therapies focusing on G9a inhibition alone.

As seen above, increasing although relatively sparse evidence suggests a role for G9a as a potential epigenetic molecular target in at least some types of adult and pediatric brain cancers, namely GBM and MB, in addition to NB, a neural tumor that can rarely occur intracranially. To our knowledge, to date, there is no evidence of a role for G9a in other brain tumor types, such as ependymoma and meningioma. Several G9a inhibitors have been put forward as potential anticancer agents, and research efforts have been successful in developing effective small molecule agents. Compounds tested in experimental brain tumor models include molecules based on different chemical scaffolds, including diazepinquinazolin-amines, benzimidazoles, natural products of chitin, and others. [89,90,91]. UNC0638 and other inhibitors are examples of a class of substrate competitive inhibitors, which directly occupy the binding site of the histone to G9a and specifically bind the substrate site in G9a, whereas other compounds constitute a second class of agents that act as competitive inhibitors of the S-adenosyl-methionine (SAM) cofactor [89]. Molecular docking experiments have identified compounds, including ninhydrin, naphthoquinone, cysteamine, and disulfide cysteamine as candidate G9a and GLP inhibitors [92]. In spite of these considerable advances in drug discovery, to date G9a inhibitors have not been evaluated in clinical trials [93,94]. Further research should increase our understanding by focusing on identifying the possible differential sensitivities of distinct brain tumor molecular subgroups and subtypes to G9a inhibition, as well as comparing different available inhibitors, including dual inhibitors, in brain tumor models, in addition to evaluating combinations of G9a inhibitors with other epigenetic or non-epigenetic anticancer agents.
